# Diving into the fish pathology of an important commercial fish species: the case of the European hake (*Merluccius merluccius* Linnaeus, 1758) in the northwest Mediterranean Sea

**DOI:** 10.1111/jfb.15940

**Published:** 2024-09-19

**Authors:** Laura Muns‐Pujadas, Maria Constenla, Sara Dallarés, Francesc Padrós

**Affiliations:** ^1^ Departament de Biologia Animal, de Biologia Vegetal i d'Ecologia Universitat Autònoma de Barcelona Barcelona Spain

**Keywords:** European hake, gross pathology, histology, histopathology, juvenile fish, parasites

## Abstract

The gross pathology and the histopathological alterations identified in juvenile specimens of the European hake (*Merluccius merluccius*), one of the most important target species of commercial fisheries in the northwest Mediterranean, are described. A qualitative and semi‐quantitative histological approach was performed in specimens from 2007 and 2019. Prevalence and mean intensity of parasites and histopathological changes were calculated in both years. No macroscopic alterations were found in any organ but several parasites (e.g., copepods, nematodes, digeneans) were detected. Microscopically, alterations identified in gills included foci of inflammation and hyperplasia (present in 77.38% of hakes from both years), extensive hyperplasia (33.33%), and inflammation (16.65%) that were potentially related to the mechanical effects of monogeneans and copepods, cysts of unknown etiology (62.69%), and lamellar inflammation associated to *Aporocotyle spinosicanalis* eggs (8.33%). Granulomas and inflammatory focus were detected in the liver, spleen, and stomach, apparently associated with the presence of nematodes. Coelozoic myxosporean parasites were detected within the renal tubules (66.66%). Most of the pathologies detected were similar to those described in other gadoid species (i.e., *Gadus morhua*) and were usually related to the presence of ecto and endoparasites. The potential impact of parasites on the health of this fish species is discussed for improving the knowledge and management of these valuable fishing stocks.

## 1 INTRODUCTION

The European hake (*Merluccius merluccius*, Linnaeus, 1758) (henceforth: hake) is a demersal fish species widely distributed in the Mediterranean Sea (Oliver & Massutí, 1995) and the third most valued species of commercial fisheries in the Mediterranean (FAO, 2020). This species occupies a wide bathymetric distribution range (from 20 to 1000 m) that is size‐dependent. Hence, juveniles mainly inhabit the continental shelf (<150 m) while larger specimens live on the upper slope (150–350 m) (Maynou et al., 2003). On the Catalan coast, where the continental shelf is the predominant fishing ground for this species, the hake juveniles are of particular importance. In 2022, their catches totaled 873.24 tons, representing 11.30% of the total captures of bottom trawling (Institut Català de Recerca per a la Governança del Mar, 2023). As individuals from this size range (<30 cm) are responsible for maintaining the population and ensuring the sustainability of the resource, the evaluation of the status and the health of this important fish stock in this area is critical for managing and conserving this species.

Although diseases occur as a normal process in any terrestrial and aquatic animal population, both infectious pathogens together with environmental conditions may compromise the health of the aquatic animal communities (Johansen et al., 2011). Fish diseases can induce many different types of alterations in the different organs and systems, compromising the functionality and viability of the organisms. As a response, these organs and systems can manage to react sometimes with a characteristic pattern and usually with the involvement of the immune system, to compensate, repair, and recover, but sometimes the organism is unable to cope with these changes. When this happens, there is a progressive deterioration of the condition that can even cause the death of the fish (Roberts, 2012).

Morphological assessment techniques such as histopathology are very useful and wide‐cope techniques to detect and evaluate diseases and pathological conditions in aquatic organisms and specifically in fish populations (Wolf et al., 2015). They can reveal subtle changes or alterations in cells or tissues that may not be apparent during gross pathology examination (Chapman et al., 2021; Eiras et al., 2008), as well as the detection of potential causative agents (Costa, 2018). In fish histopathological studies, the main target organs used are the gill and liver due to the direct exposure with the water as well as its relevant role in respiration and osmoregulation and as responsible for the biotransformation and elimination of noxious substances (Bernet et al., 1999), respectively. However, other organs such as the digestive tract, gonads, spleen, and kidney are also very important, as are target organs for many external agents (i.e., parasites), and useful for assessing the individual's or stock global health status (Wolf et al., 2015).

Parasitic infections are particularly widespread among wild fish (Feist & Longshaw, 2008), and the combined use of parasitological and histopathological techniques is especially useful to assess their presence, associated parasitological descriptors as well as the potential damage to the host. Although many of them in normal conditions have low or negligible impact on fish, in some cases their significance may be enhanced depending on the infection intensity and severity, the host response and other coexisting factors, such as the condition of the fish or environmental effects (Feist & Longshaw, 2008; Sures & Nachev, 2022). As examples of responses, most endoparasites are typically encapsulated by host reaction in internal organs while ectoparasites usually elicit severe damage due to the mechanical damage caused by their attachment (i.e., in the gills) (Feist & Longshaw, 2008). Hence, infected fish may experience reduced growth, impaired organ function, reduced reproduction success, increased susceptibility to other diseases, or increased mortality, which can lead to population declines and ecosystem‐level effects (Ryberg et al., 2020).

Accurate histopathological diagnosis requires proper sample handling and expert interpretation of histologic slides to prevent misdiagnosis and misinterpretation (Wolf et al., 2015). Various approaches exist for assessing histopathological features, with qualitative analysis forming the basis for understanding new lesions and patterns of histological alterations in aquatic organisms (Costa, 2018; Costa et al., 2009). Current research is mostly focused on establishing cause–effect relationships between histological changes and contamination using weighted indices (Bernet, 1999; Costa et al., 2009; Cuevas et al., 2015; Saraiva et al., 2015). Although diseases in wild fish are often defined by atypical events (e.g., temporary pollution sources or epizootics), most of them are nonspecific and occur naturally (Bergh, 2007). Hence, a descriptive overview of the potential fish diseases is fundamental for evaluating future changes, sometimes complemented by a semi‐quantitative approach based on the severity of the lesion (Costa, 2018).

The study of the causative pathogens and diseases affecting gadoid fish species has been in the spotlight given their interest and particularly cod as farmed species (Bricknell et al., 2006). Specifically, viruses, bacteria, and parasites have been the focus as disease‐causing agents in both wild and cultured *Gadus morhua* Linnaeus, 1758 (Birkbeck et al., 2011; Bricknell et al., 2006; Samuelsen et al., 2006). In these studies, a macroscopic and histological description of tissue alteration was performed in those fish that started to die or presented signs of infection (i.e., altered swimming behavior, reduced weight, emaciated appearance) to evaluate the extension, severity, and intensity of the lesions caused by specific pathogens such as the bacteria *Vibrio anguillarum* and *Francisella* spp. and the parasitic copepod *Lernaeocera branchialis*. In the particular case of hake, most of the histopathological studies performed in this species attempted to detect tissue alterations associated with contaminants (Cuevas et al., 2015; Marigómez et al., 2006). However, most of the alterations and lesions identified in those studies such as inflammatory changes, atrophy, necrosis, apoptosis, neoplasms, foci of cellular alteration, and melanomacrophage centers, could not be related only to pollutants' effects, but to other biological confounding factors. Moreover, some histological studies based on the reproductive biology of hake have been performed in the Mediterranean Sea mostly on females (Candelma et al., 2021; Carbonara et al., 2019) and recently also in males (Mascoli et al., 2023), so biological factors such as age, sex or reproductive stage can introduce further variability in the interpretation of the changes. Yet, studies focused on the diseases that may occur naturally in hake populations are still lacking.

Thus, the present study aims to provide insight into the identification and description of the gross pathology, parasites, and histopathological alterations found in different target organs from juvenile specimens of *M*. *merluccius* captured on the continental shelf off the coast of Barcelona, in the northwest Mediterranean Sea. Moreover, a qualitative and semiquantitative histological approach was performed in specimens from 2007 and 2019 to detect potential temporal histopathological changes.

## 2 MATERIALS AND METHODS

### 2.1 Study area and data collection

A total of 39 *M*. *merluccius* specimens were collected on board commercial fishing trawlers on the continental shelf off the coast of Barcelona (northwest Mediterranean) in summer 2007 (*n* = 18, 22.37 cm SL) and 2019 (*n* = 21, 17.31 cm SL) (Table 1), within the framework of the BIOMARE (Spanish Ministry of Science and Innovation) and PLASMAR (Spanish Ministry of Science, Innovation and Universities) projexts, respectively. After capture, fresh fish were externally examined for the presence of macroscopic alterations and immediately fixed in toto in 10% buffered formalin after performing an abdominal incision to allow proper fixation of internal organs, for further analysis.

**TABLE 1:** Study area data (season, depth in m, latitude and longitude) for each sampling year, including the number (*n*), mean standard length (SL) followed by the standard deviation (SD) in cm, percentage of immature and mature individuals, and the sex ratio (M:F) from *Merluccius merluccius* specimens collected in 2007 and 2019.

	2007	2019
Season	Summer	Summer
Depth (m)	62.4	52.5
Latitude	41°25′22.05″N	41°19′45.72″N
Longitude	2°20′55.29″E	2°17′8.279″E
*n*	18	21
SL (cm)	22.37 (3.10)	17.31 (1.35)
Immature (%)	44.44	80.95
Mature (%)	55.56	19.05
Sex ratio (M:F)	1:9	1:4

### 2.2 Gross pathology and parasitological assessment

Fixed fish were externally examined, dissected, and excised organs were examined under a stereomicroscope for the presence of macroscopic alterations and/or parasites. All parasites found were collected, counted, and stored in 70% ethanol. For identification, platyhelminths and acanthocephalans were stained with iron acetocarmine, dehydrated through a graded ethanol series, cleared in clove oil, and examined as permanent mounts in Canada balsam. Nematodes were cleared and examined as semipermanent mounts in glycerine. Parasites were identified to the lowest taxonomic level whenever possible (Gibson et al., 2002; Kabata, 1979; Moravec, 1994). Parasite prevalence (P) and mean intensity (MI) were calculated according to Bush et al. (1997) for the total of parasite species recovered from each year.

### 2.3 Histopathological assessment

During the dissection of each fixed individual, a portion of liver, spleen, and gills from the left side were processed by routine paraffin histology. Moreover, from specimens collected in 2019, a portion of gonads, kidney, stomach, and intestine were also processed. Each organ was sectioned at 4–5 μm and stained with hematoxylin and eosin. All the stained slides were thoroughly screened and examined under the microscope for the detection and identification of specific disorders. Some liver sections were additionally stained with periodic acid Schiff (PAS). Images were taken with a Leica camera model CTR5000 connected to a Leica DM500B microscope.

The prevalence of each histopathological alteration in hake populations (2007 and 2019) was calculated from one section of each organ. Specifically, in the gills and for an accurate assessment of the alterations, histological evaluation was based on filaments that were sectioned in the standard orientation and thus lamellae were of equal length on both sides of the filament. The mean intensities of each histological alteration found in each organ with prevalences >15% were calculated from the average intensity values of the specific histological alteration in each individual, excluding those fish without the infection (Table 2). Particularly in gills, a semiquantitative analysis was applied for alteration foci (focal inflammatory response and epithelial hyperplasia with lamellar fusion). In these cases, from each gill section, the number of lamellae affected by alteration foci was counted and scored as mild (<4 lamellae per filament), moderate (4–10 lamellae), or severe (>10 lamellae). In addition, to evaluate the intensity of the infection by *Aporocotyle spinosicanalis*, the total number of eggs within each affected filament was counted and scored as mild (1) (<5 eggs per filament), moderate (2) (5–15 eggs per filament), or severe (3) (>15 eggs per filament) (Table 2). For the alterations detected in the rest of organs, three fields of view at 100× or 200× of magnification (0.91 mm^2^/screen and 0.24 mm^2^/screen, respectively), were randomly selected and counts of the number of each histopathological alteration were performed on each section. Densities of these alterations (number per square millimeter) were calculated for each of the three fields and mean values were given for each specimen (Table 2).

**TABLE 2:** Prevalence (%) and mean intensity (MI) followed by range values of the parasites recovered from *Merluccius merluccius* in 2007 and 2019. Prevalence (%) and mean intensity (MI) or mean density (MD) in terms of number/mm2 followed by the standard deviation (SD) of the histopathological alterations are also displayed.

		2007		2019	
Parasitology	Location	P%	MI (min–max)	P%	MI (min–max)
Myxozoa^a^	K	X	X	66.66	X
Monogenea					
*Anthocotyle merluccii*	G	–	–	14.28	1.00 (1–1)
Digenea					
*Aphanurus virgula*	S	5.55	1.00 (1–1)	–	–
*Aporocotyle spinosicanalis*	H	22.22	1.25 (1–2)	–	–
*Lecithaster gibbosus*	I	–	–	19.04	2.00 (1–3)
*Lecithocladium* sp.	S	–	–	14.28	1.67 (1–3)
*Hemiurus* sp.	S	5.55	1.00 (1–1)	23.81	1.40 (1–2)
*Ectenurus lepidus*	S	–	–	9.52	2.00 (2–2)
*Prosorhynchus crucibulum*	S	5.55	1.00 (1–1)	–	–
*Parahemiurus merus*	I	–	–	4.76	1.00 (1–1)
Cestoda					
*Clestobothrium crassiceps*	S, I	22.22^B^	1.75 (1–3)	71.43^A^	1.40 (1–2)
*Scolex pleuronectis*	I	5.55	2.00 (2–2)	–	–
Nematoda					
*Hysterothylacium aduncum*	S, I	11.11^B^	2.00 (1–3)	52.38^A^	1.55 (1–2)
*Hysterothylacium fabri*	S, I, L, G, PT	22.22	1.00 (1–1)	19.04	2.00 (1–4)
Copepoda					
*Lernaeocera* sp.	G	11.11	2.50 (1–4)	14.28	1.00 (1–1)
*Neobrachiella* sp.	G	–	–	9.52	1.00 (1–1)

Histopathology	Location	P%	MI (SD)	P%	MI (SD)
Alteration foci	G	83.33	1.16 (0.43)	71.43	1.33 (0.62)
Extensive hyperplasia	G	33.33	2.33 (1.21)	33.33	4.29 (2.36)
Extensive inflammation	G	–	–	33.33	1.14 (0.38)
CUEs	G	77.77^A^	1.79 (0.80)	47.62^B^	1.50 (0.85)
*Aporocotyle spinosicanalis* eggs	G	16.66	1.63 (0.69)	–	–

		P%	MD (SD)	P%	MD (SD)
Granuloma	L	5.55^B^	X	19.05^A^	0.27 (0.18)
	Sp	–	–	4.76	X
	S	–	–	23.81	0.22 (0.18)
MMCs	Sp	66.66^A^	7.81 (2.91)	4.76^B^	X
	K	–	–	4.76	X
Aggregated macrophages	L	66.66^A^	5.30 (5.15)	4.76^B^	X
	Sp	5.55^B^	X	52.38^A^	18.68 (14.77)
Inflammatory focus	L	11.11	X	9.52	X
Rodlet cells	S	X	X	47.62	X

*Note*: Keys for locations within host: G, gills; H, heart; I, intestine; K, kidney; L, liver; Sp, spleen; S, stomach; PT, perivisceral tissues. Keys for histopathological alterations: CUEs, cysts of unknown etiology; MMCs, melanomacrophage centers. Different superscript letters indicate significant differences between years in prevalence. Dashes indicate absence of the parasite or histopathological alteration. X, non‐available data.

^a^ Data from histological analyses.

### 2.4 Data analysis

All variables were tested for normality and homoscedasticity using the Shapiro–Wilk test and the Levene's test, respectively. Data distribution was also plotted for visual assessment. To detect differences in the prevalence of parasites and histopathological alterations between years, the chi‐squared test was applied. Likewise, differences between years in mean intensity of parasites and histopathological alterations were tested using Student's *t*‐test (assuming non‐equal variances) or Wilcoxon rank sum (when normality conditions were not accomplished) tests. Moreover, to identify significant differences in the prevalence and mean intensity of parasites depending on the fish sex (immature, mascle, or female), chi‐squared and Kruskal–Wallis tests were performed for each year, respectively.

## 3 RESULTS

### 3.1 Gross pathology and parasitological observations

**FIGURE 1:**
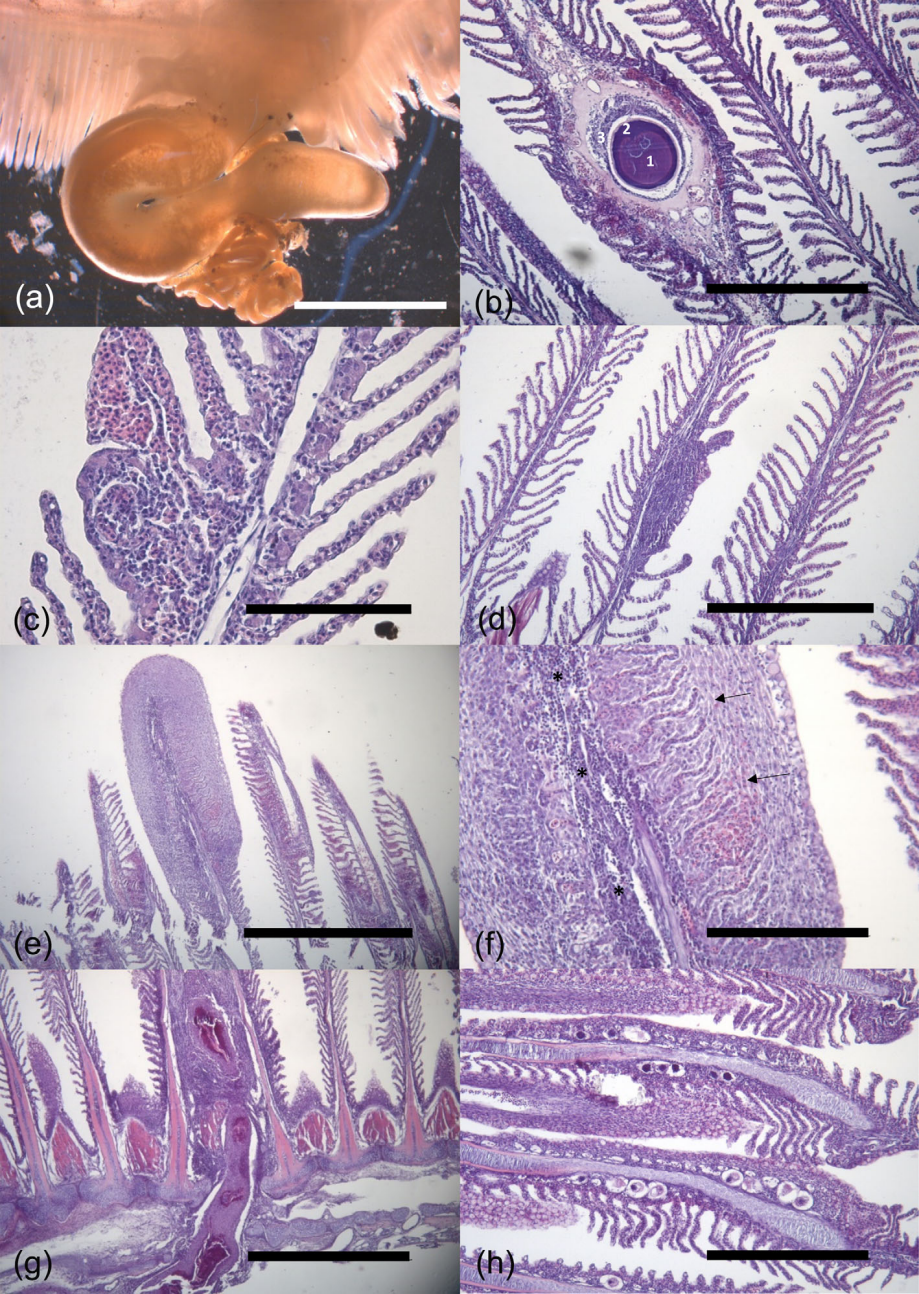
Histological (H&E stain) gill sections in *Merluccius merluccius*. (a) A female of the copepod *Lernaeocera* sp. with the head embedded in the gill arch. Note coiled egg sacs (arrow). Scale bar = 500 μm. (b) Cysts of unknown etiology in a gill filament. An eosinophilic central core (1) surrounded by acellular basophilic material of varying thickness (2) is recognized. An inflammatory response of macrophages and connective tissue is observed surrounding the cyst (3). Scale bar = 400 μm. (c) Mild, (d) moderate, and (e) severe alteration foci. Note the presence of aggregation of immune cells infiltration in the gill tissue. Scale bar = 100, 400, and 800 μm, respectively. (f) Higher magnification of (e). Note the presence of inflammatory cell infiltration of lymphocytes inside the filament with hyperplasia (asterisks) and a proliferation of cell activity leading to an increase in the thickness of the lamellar epithelium (arrows). Scale bar = 200 μm. (g) Possible site fixation of a copepod infecting the gill causing an inflammatory response and extended hyperplasia involving two filaments. Scale bar = 800 μm. (h) Gill filaments with some *Aporocotyle spinosicanalis* eggs with different degree of development. Scale bar = 400 μm.

No macroscopic alterations were found in any examined organ except on the gills, in which the only alterations detected were small whitish spherical nodules of about 200–300 μm in diameter located within the gill filament, which histologically corresponded to cysts of unknown etiology (CUEs). *Lernaeocera* sp. specimens were also detected attached to the branchial gill arch in 12.82% of the hakes examined from both years (Figure 1a). Most of the individuals from 2019 were immature but more than half of the specimens from 2007 were mature, with female the predominant sex (Table 1). The gonadal morphology was consistent with the description provided by ICES (2007).

A total of 66.66% of the hakes sampled in 2007 and 100% of the hakes sampled in 2019 were infected by at least one parasite. Gills and intestines were found to be the most parasite‐infested organs. A total of 111 parasites belonging to seven different taxa were identified in different organs (Table 2). Among the recovered taxa, digeneans were the main contributors to species richness and mean intensity values. The nematode *Hysterothylacium aduncum* and the cestode *Clestobothrium crassiceps*, both found in the stomach and intestine, presented a significantly higher prevalence (chi‐squared, *ꭓ* ^2^ = 7.43, *p* = 0.006 and *ꭓ* ^2^ = 9.39, *p* = 0.002, respectively) in fish captured in 2019 compared to 2007 (Table 2). In contrast, the digenean *A*. *spinosicanalis*, located in the heart, was only detected in specimens captured in 2007(Table 2). No significant differences in the mean intensity of parasites were detected between years (*p* > 0.05). Similarly, there were no significant variations in the prevalence and mean intensity of parasites among immature, male, and female fish from each year (*p* > 0.05).

### 3.2 Histological and histopathological findings

#### 3.2.1 Gills

About 89% of the filaments examined displayed a normal morphology but the remaining 11% of the filaments presented at least one alteration, regardless of the type, size or extension of the alteration (Table 2).

CUEs were one of the most frequent alterations observed in gills (Table 2). The number of CUEs found in a section of a single gill arch ranged from one to four. CUEs consisted of a homogeneous core of acellular and eosinophilic material surrounded by a thin layer of basophilic material usually in central position in the filaments (Figure 1b). Other layers of variable thickness were sometimes observed surrounding the CUE, which could be composed by an inflammatory response of macrophages and connective tissue, capillary vessels, and/or cartilage. The presence of CUEs was significantly higher in individuals from 2007 (chi‐squared, *ꭓ* ^2^ = 4.88, *p* = 0.03) (Table 2).

Other recurrent findings detected in gill's filaments were telangiectasia identified as blood expansions of lamellar capillaries and inflammatory cell infiltration, which was generally composed of lymphocytes and granular cells. This inflammatory response was found as discrete foci typically affecting the interlamellar spaces of two to 15 lamellae (Figure 1c–f), but also within the filament epithelium. Moreover, more extensive areas of inflammation were also detected affecting mostly the central core of the whole filament and to a lesser extent expanding to the interlamellar spaces. Epithelial lamellar hyperplasia with fusion of adjacent lamellae was also associated with discrete foci (affecting from two to more than 10 lamellae) (Figure 1c–f) or even extensive (affecting one or more entire lamellar epithelial cells), leading to an increase in the total thickness of the gill epithelia. No significant differences between years were detected in either the prevalence or the mean intensity of alteration foci and extensive inflammation and hyperplasia (*p* > 0.05) (Table 2). These lesions were often observed associated to the attachment of the copepod *Lernaeocera* sp., which also caused extensive inflammation and hyperplasia in the adjacent filaments (Figure 1g). In some cases, areas with strong inflammatory reaction and necrosis were identified in the gill arch. Lamellar fusion and inflammation were also detected, but not always, associated with the presence of embolized eggs with different degrees of development of *A*. *spinosicanalis* in the afferent arteries of gill filaments (Figure 1h). Eggs were observed in low prevalence only in fish from 2007 and displayed low mean intensity values (Table 2).

#### 3.2.2 Liver

**FIGURE 2:**
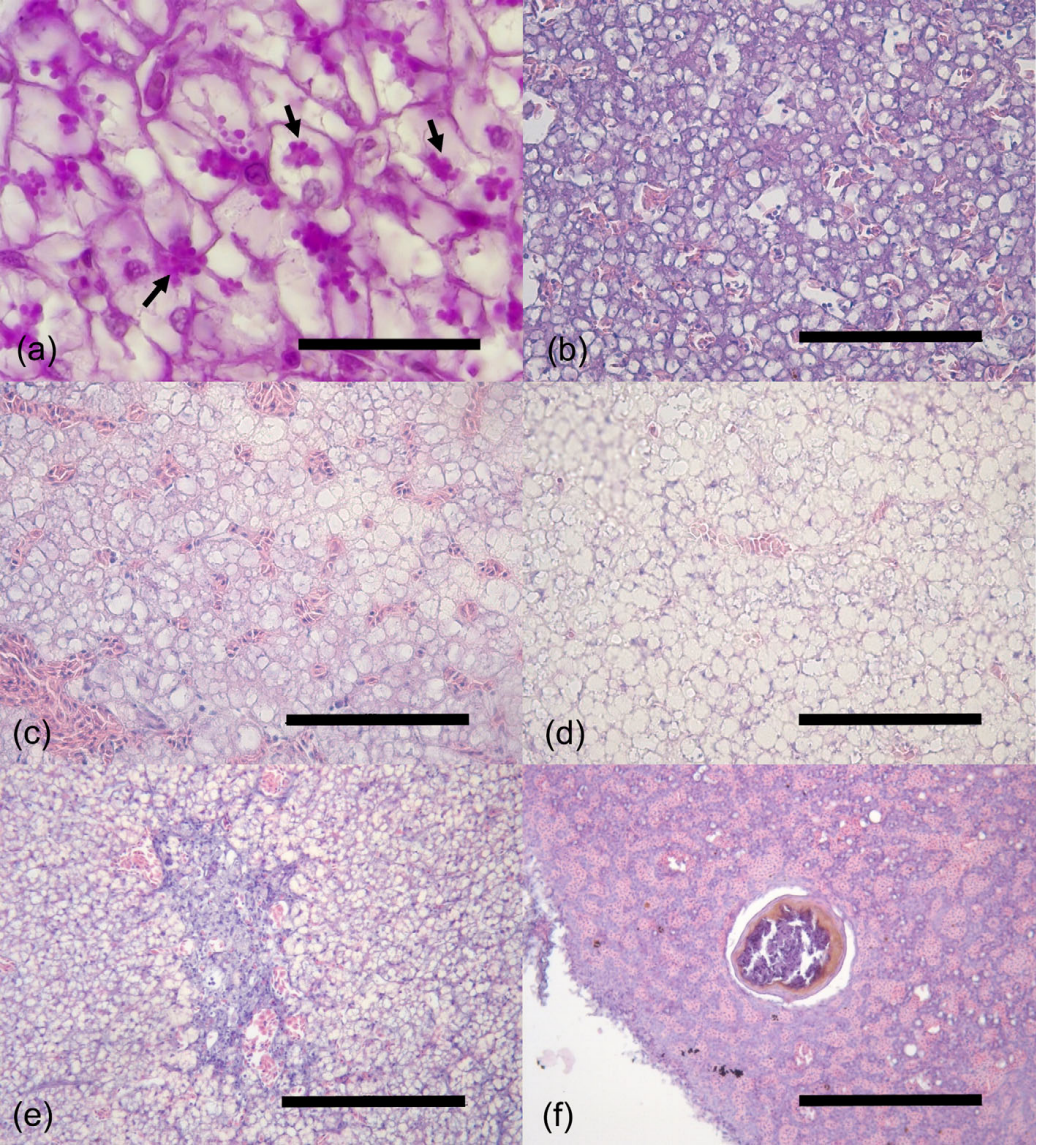
Histological (H&E stain) sections of hepatic parenchyma morphologies and alterations of *Merluccius merluccius*. Scale bar = 100 μm. (a) Hepatocytes with glycogen stained by periodic acid Schiff (PAS) stain (arrows). Scale bar = 30 μm. (b) Lipidic metamorphosis I with small and non‐aggregated lipid droplets within the hepatocytes, barely displacing the nucleus and cytoplasm towards the periphery of the cell. (c) Lipidic metamorphosis II with different lipid droplet sizes, some of them aggregated in larger droplets, displacing the nucleus and the basophilic cytoplasm to the periphery of the cell, making it difficult to discern the limits of the cell. (d) Lipidic metamorphosis III with large and abundant lipid droplets merging together within the hepatocytes totally displacing the nucleus to the periphery of the cell. Basophilic cytoplasm was totally absent, the cellular limits were diffused and completely undistinguished. (e) Inflammatory focus in the hepatic parenchyma. Scale bar = 200 μm. (f) A granuloma in the hepatic parenchyma. Scale bar = 200 μm.

A wide range of diverse hepatic parenchyma morphologies were identified in different specimens and clearly associated with the amount of reserve substances (i.e., glycogen or lipid) stored within the hepatocyte cytoplasm (Figure 2a–d). The round‐white droplets most probably represented areas previously occupied by lipids that were removed during tissue processing. According to the degree and pattern of lipid droplets accumulated within the hepatocytes (termed as lipidic metamorphosis), three different categories were identified: lipidic metamorphosis I consisted of simultaneous areas of hepatocytes without reserve substances and hepatocytes displaying scarce lipid droplets within the cytoplasm (Figure 2b); in lipidic metamorphosis II, lipid droplets occupied more than 50% of the cytoplasm (Figure 2c); and lipidic metamorphosis III was the category with the most abundant lipid droplets accumulated within hepatocytes (Figure 2d).

Regarding the histopathological changes detected in the liver, different alterations found in the hepatic sections consisted of focal inflammatory reactions mainly composed of lymphocytes sometimes identified in the vicinity of the sinusoids (Figure 2e), and granulomatous inflammatory responses of the host in the hepatic parenchyma and serosa. These granulomatous inflammations were present as discrete spherical granulomas consisting primarily of a concentrically arranged mixture of mononuclear cells (mainly probably macrophages and lymphocytes) and/or fibroblasts surrounding a necrotic center sometimes associated with melanin, which could correspond to either the lesion caused by a pathogen (i.e., a nematode) or the degraded form of the pathogen itself (Figure 2f). Some apparently viable nematode larvae were seen mostly in the hepatic serosa but also in the parenchyma. Also, isolated or aggregated macrophages, identified as simple units of two or three cells containing brownish (lipofuscins or ceroid) or blackish (melanin) pigments, were observed. A significantly higher prevalence of granulomas in livers of fish from 2019 and a higher presence of macrophage aggregates in specimens from 2007 were detected (chi‐squared, *ꭓ* ^2^ = 7.07, *p* = 0.008 and *ꭓ* ^2^ = 16.71, *p* < 0.001, respectively).

#### 3.2.3 Spleen

**FIGURE 3:**
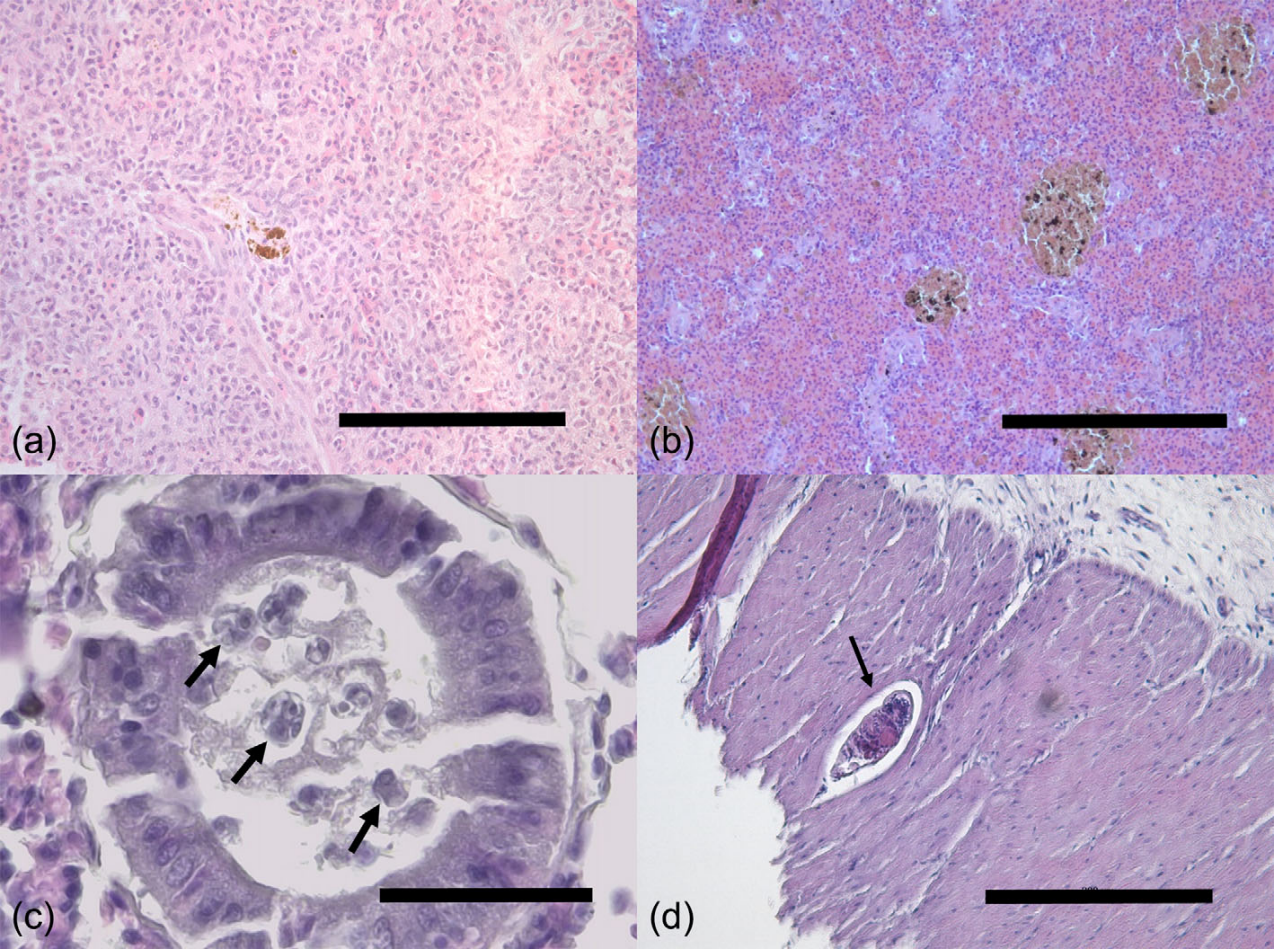
Histological (H&E stain) sections of alterations identified in different organs of *Merluccius merluccius*. (a) Macrophages aggregates containing ceroid pigment in the splenic tissue. Scale bar = 100 μm. (b) Melanomacrophagic centres (MMCs) within the splenic parenchyma. Scale bar = 200 μm. (c) A renal tubule with coelozoic myxosporean parasites (arrows). Scale bar = 30 μm. (d) A non‐encapsulated digenean metacercaria (arrow) in the stomach muscular layer without causing any host reaction. Scale bar = 200 μm.

The splenic tissue was characterized by the presence of scattered aggregations of macrophages (Figure 3a) and melanomacrophage centers (MMCs) containing melanin or lipofuscin pigment. These MMCs were in general small‐sized (~66 μm in diameter) with an irregular shape containing a dark‐brown pigment and were detected as a distinctive grouping of more than five melanomacrophages displaying an organized structure homogeneously distributed throughout the splenic tissue (Figure 3b). A few cases of granulomas with the same structure as found in liver were also detected in 4.76% of the specimens. A significantly higher prevalence of MMCs in individuals from 2007 but a significantly lower prevalence of aggregated macrophages compared to those from 2019 was detected (chi‐squared, *ꭓ* ^2^ = 16.71, *p* < 0.001 and *ꭓ* ^2^ = 15.25, *p* < 0.001, respectively). On the contrary, a higher density in number of aggregated macrophages in individuals from 2019 was found compared to the density of MMCs from individuals collected in 2007.

#### 3.2.4 Kidney

The histological assessment of the renal tissue revealed the presence of pre‐sporogonic stages of coelozoic myxosporean parasites in the lumen of the renal tubules from 66.66% of the hake, but no direct tubular degeneration, damage, or host reaction was detected in the tubules with parasites (Figure 3c). In certain cases, lymphocytic infiltration throughout the renal tubules was detected as well as mineralized or dark material accumulated within degenerated tubules and, in these cases, MMCs were also present nearby.

#### 3.2.5 Digestive tract

Slight alterations were revealed from the histological sections of the stomach and intestine such as hyperhemia identified as an increase of blood cells within the intestinal mucosa in 9.67% of the individuals. Large numbers of rodlet cells were present in the stomach mucosa from 47.62% of hake from 2019 (Figure 3d). In 23.81% of the individuals, granulomas with the same structure as described above in the liver were present in the submucosa‐lamina propria, mostly associated with the presence of degraded nematode larvae and with a scarce inflammatory reaction surrounding the granuloma. Likewise, non‐encapsulated digenean metacercariae were detected in 4.83% of hake from 2019 located in different layers from the stomach without apparent host reaction. Digeneans, nematodes, and cestodes were present within the intestinal lumen but no histological alterations associated with their presence were detected. However, a single pathological finding was found associated to the attachment of the scolex of the cestode *C*. *crassiceps* to the intestinal mucosa.

#### 3.2.6 Gonads

In all histological sections from 2019, gonads displayed early gametogenic stages, and in 80.95% of the individuals, gender could not be differentiated by microscopical characteristics since they were immature. Instead, four sections of gonads could be distinguished: ovaries showed compact ovigerous lamellae with germline cysts containing oogonia and oocytes in primary stage with basophilic ooplasm surrounded by connective tissue and a thin ovarian membrane. Likewise, testicles were in an early spermatogenic stage, characterized by the predominantly presence of spermatogonia and primary spermatocytes. No histopathological alterations were detected in the gonadal tissue.

## 4 DISCUSSION

Fish health assessment studies are essential as integral components of environmental evaluation and conservation of natural resources. Understanding the main diseases that important commercial fish species may present is an indispensable tool for monitoring ecosystem health. The present study offers insights into the main pathologies of *M*. *merluccius* juvenile populations in the Catalan coast, a particular area of the northwest Mediterranean Sea, as a first step in gaining knowledge of the overall health status of this species.

Although histopathology is an effective diagnostic technique to detect changes in the normal structure of any organ, caution should be taken as artifacts are easily produced during sampling collection, preservation, and processing. As Wolf et al. (2015) exhaustively reported, the histological preparation of the gills, along with the kidney and liver, can be technically challenging. Moreover, vascular changes should be interpreted cautiously, as their manifestation can be imitated by artifacts caused during the process of collecting samples. In particular, telangiectasia was detected in almost every examined gill section from this study, which typically is resolved via lamellar thrombosis or accumulation of erythrocytes in some gill areas. Thrombotic evidence indicates the prior existence of telangiectasia in gills before necropsy, while a lack of thrombotic changes implies acuteness in lesion duration and questionable pathologic significance (Wolf et al., 2015). Hence, due to the absence of thrombosis evidence and since telangiectasia and hyperhemia are alterations induced by trawling, they were not considered pathological findings.

Gadoids have been stated to be hosts of a broad nature of parasites due to the wide range of geographical areas and habitats they occupy (Bricknell et al., 2006), as is well represented by the diverse parasite fauna of *G*. *morhua* (Hemmingsen & MacKenzie, 2001) and by the parasite community of *M*. *merluccius* revealed in this study, which has also been well described in the literature (Carrassón et al., 2019; Ferrer‐Maza et al., 2014). Macroscopic lesions that occur in gadoid fish species are not commonly reported (Feist et al., 2004), but some of them have been mainly associated with ecto and endo parasites. Some metazoan endoparasites are normally found freely in the lumen of the digestive tract without causing any significant pathogenic effect on the host (Hemmingsen & MacKenzie, 2001), but some of them can be macroscopically found encysted by the host. For example, white, opaque, and spherical cysts of microsporidians and Pseudophyllidean cestodes have been identified in the muscle and stomach of *Micromesistius poutassou* Risso, 1827, respectively, but no host reaction, apart from the encapsulation, was detected (MacKenzie, 1981). In heavily infected fish, intensive host reactions can be developed and, in many cases, with the production of melanin pigments by different cell types. In fact, multilayered masses of epidermal cysts of *Cryptocotyle*‐like heterophyid metacercaria are related to skin melanosis in *G*. *morhua* (Duflot et al., 2021). In some reports, protozoan infections have been reported to be more pathogenic to their host (Hemmingsen & MacKenzie, 2001). Indeed, in heavy coccidian infections in the liver of *M*. *poutassou*, the hepatic tissue was replaced by oocysts reducing fish condition (Abollo et al., 2001). Although coccidian infections have been described in many fish species, including *Merluccius* spp. (Morrison & Marryatt, 1990), no coccidians were found in this study. Other reported macroscopic alterations have been related to other causative agents such as bacterial infections or pollutants, as it is the case for dermal ulcers detected in *G*. *morhua* (Borucinska & Morka, 2016). The lack of macroscopic alterations (i.e., neoplasms) revealed from present findings has also been reported in other studies from the same species (Cuevas et al., 2015; Marigómez et al., 2006). This could be explained by the fact that most of the examined specimens were immature. In fact, age has been linked with disease susceptibility in most aquatic organisms, but species genetics, environmental conditions or anthropogenic pollutants should also be considered (Stentiford et al., 2010). Indeed, it has been reported in the flatfish dab *Limanda limanda* Gottsche, 1835 that grossly visible diseases such as epidermal hyperplasia, ulcerations of the skin, and hepatic neoplasms do not appear in noticeable prevalence until the age of 4 years (Stentiford et al., 2010). Moreover, the difficulty in encountering gross lesions in juvenile hake specimens could be limited to the sampling methodology used as some macroscopic alterations may be obscured in fixed specimens, but may be also related to the fitness of the individuals, given that in wild conditions those individuals with reduced fitness due to diseases are likely to not survive and therefore fewer chances exist to be caught off commercial fisheries (McVicar, 1997).

Regarding histopathological alterations, the gills appeared to be the most affected organ due to the close contact with water and particular tropism for gills for many diseases. Depending on the type and severity of the stressor and length of exposure, gills can respond in a wide variety of ways. In particular, most of the lesions identified in gill filaments and lamellae (foci or extensive inflammation and hyperplasia) of the present study were probably mostly related to the mechanical effects caused by the presence of monogeneans, copepods, and blood fluke eggs.

Monogeneans can cause focal damage mainly due to their attachment to the gills with their posterior adhesive organs, although in some cases they can also harm the gill structure by their feeding activity. The polyopisthocotylean *Anthocotyle merluccii* has been identified in this study and is also well‐reported in *M*. *merluccius* in the northwest Mediterranean Sea (Carrassón et al., 2019; Ferrer‐Maza et al., 2014) and other species from the genus *Merluccius* (MacKenzie & Longshaw, 1995). This monogenean has four pairs of clamps and uses its anterior‐most enormously developed pair of clamps to grasp the inner border of the filament and the three small remaining pairs of clamps attach to secondary lamellae (Llewellyn, 1956). In this study its pathogenic significance is not important given the low prevalence of the parasite and the low intensity of inflammation affecting the lamellae.

In the case of the copepod *Lernaeocera* sp., the extensive tissue necrosis and associated inflammatory response observed in the attachment site from the gill arch, and the extensive epithelial hyperplasia identified in the gill filaments surrounding the attachment site, were most probably caused by the mechanical effect triggered by the presence of the external part of the body of the parasite. In fact, it was reported that *Lernaeocera* uses its thoracic holdfast to penetrate the gill arch and reach the main arteries (Kearn, 2005) and even cardiac structures. These observations are comparable to those related for other similar parasitic copepod species. For example, the damage caused by the attachment of *Lernaeocera branchialis* to the gills of *G*. *morhua* (Behrens et al., 2014; Smith et al., 2007), or by *Lernanthropus radiatus* in the gills of Black seabream *Spondyliosoma cantharus* Linnaeus, 1758 (Lovy & Friend, 2020). Indeed, copepods are of particular relevance due to their potentially pathogenic effect as they may provoke severe damage, depending on their size, by grasping, anchoring, and feeding in the gill arch and filament (Colorni & Padrós, 2011). However, in this study, the inflammatory reaction against copepods may not suppose any vital threat to fish condition, probably due to the low number of copepods recovered and the low intensity of infection.

Likewise, the presence of the blood fluke *A*. *spinosicanalis* recovered in 2007 should be considered as blood fluke parasites have been reported to be a problem in many cultured fish species as they can accumulate in large numbers inside the gills and blood vessels, obstructing blood circulation, evoking inflammatory responses, and reducing the respiratory surface (Holzer et al., 2008; Padrós et al., 2001; Shirakashi et al., 2012). In the current study, the eggs were only present within the filament arteries, indicating that they were not able to pass through the lamellae capillaries, probably due to their size. In this study, given the low prevalence, extension, and intensity of infection detected and the mild host reaction, no relevant detrimental effects on the cardiorespiratory system could be expected. In wild conditions, a combination of abiotic factors, the seasonal development of the blood fluke's life cycle as well as the length size of the host have been suggested to be important drivers that may influence the incidence of infection (Bullard & Overstreet, 2002; Holzer et al., 2008). Although the presence of both adult and eggs of *A*. *spinosicanalis* was only detected in 2007, no clear temporal patterns could be drawn. Hence, further research should be considered for this species since the incidence of infection could be significantly higher in adult individuals and consequently compromise their health condition in the future.

CUEs appeared to be a significant alteration as they were commonly found in both years. Although a significantly higher prevalence was detected in 2007, wide local variations in their prevalence may occur, as reported by MacKenzie and Longshaw (1995), in which different prevalences of CUEs were displayed among adjacent samples in *Merluccius hubbsi* Marini, 1933 and *Merluccius australis* Hutton, 1872. The morphology of the CUEs present in the gill sections was similar to those described by other authors in other fish species (Carreras‐Aubets et al., 2012; Constenla et al., 2015, 2022; Dallarés et al., 2014). Although the etiology of these structures remains unknown, it was suggested that this alteration could be related to environmental pollution (Carreras‐Aubets et al., 2012; Constenla et al., 2015), although this affirmation is still controversial due to contradictory observations (Heidel et al., 2002).

The morphology of the hepatic parenchyma and the amount of reserve substances such as lipids and glycogen present in the hepatocyte cytoplasm are subject to significant variation among fish, attributable to factors such as species, age, gender, nutritional status, seasonal changes, and developmental stage (Wolf et al., 2015). Hepatocytes store excess energy as glycogen or lipid in the cytoplasm when energy intake exceeds needs for metabolism, growth, exertion, reproduction, and so forth (Wolf et al., 2015). Although vacuolation within the hepatocytes has been reported in fish exposed to toxic substances (Zodrow et al., 2004), in many cases intracytoplasmic lipid droplets have been misinterpreted as pathological vacuolation, contributing to the confusion in the interpretation of these findings. It should be considered that hake, like other gadiform fish (i.e., *G*. *morhua*), accumulate large amounts of lipids naturally (Morrison, 1987), particularly in the liver. In the present outcomes, hepatic pleomorphism was present within individuals from the same season (i.e., summer), and although most of the livers were homogeneous, some of them presented different levels of lipid vacuolization. The propensity to store glycogen or lipid seems to vary among fish species and may be also influenced by dietary factors (Wolf et al., 2015).

In the present study, no histopathological neoplastic changes were observed in the hepatic tissue of the examined hakes, lesions frequently described in surveys on other fish species (Feist et al., 2004). In hake, high prevalence of hepatocellular nuclear pleomorphism was described after the Prestige oil spill on the Bay of Biscay (Marigómez et al., 2006), but lower prevalences were reported a few years after, indicating a recovery from the chemical spill (Cuevas et al., 2015). However, the absence of neoplastic changes in this study should not be interpreted as a lack of indication of the effects of pollution. As it was previously said, this study was focused only on hake juvenile specimens, and further research, including different ages, is needed to assess the impact of toxic pollutants from the Catalan coast on the health status of hake.

The liver was found to be the most infected organ by nematode larvae (mostly by *H*. *fabri*), which was mainly recovered in this organ during gross inspection. *Hysterothylacium* is a well‐known nematode genus that infects several gadoid fish species (Bricknell et al., 2006). Reported lesions in gadoid's livers related to the presence of *Hysterothylacium* and *Contracecum osculatum* resulted in granulomatous changes, inflammation, hemorrhages, atrophy, reduced fat content, and destruction of the hepatocytes (Behrens et al., 2023). Granulomatous reactions surrounding apparent degenerating nematode larvae were found predominantly in this study, particularly in 2019, for their presence also in the stomach and kidney. This granulomatous response had similar ultrastructural features of encapsulation as described in other gadoid fish species (Behrens et al., 2023; Dallarés et al., 2014, 2016). Granulomas are chronic inflammatory focal lesions that may be the result of the infection of bacteria, viruses, protozoa, and metazoan parasites (Ferguson, 2006; Roberts, 2012; Wickins et al., 2011) and in this case most of them seem to be associated with nematodes.

The presence of pigmented macrophages isolated, aggregated, or forming centers in both liver and spleen was noticeable in both years. Although MMCs can be considered biomarkers of environmental stress, other factors such as inflammation caused by pathogens and tissue damage, tissue repair, or even the age, size, and sex of the fish, considering the natural variability in different fish species, should be deemed more important (Carreras‐Colom et al., 2022). In the current study, variability in the intensity of MMCs was noticed among the specimens examined but was not either related to age or sex.

The histopathological changes detected in the digestive tract tended to be minimal and localized to the region of parasite infection (i.e., digeneans, cestodes, and nematodes). Although a high diversity of helminth parasites was found in the lumen of both stomach and intestine, only scattered, slight, or negligible host responses were detected. These responses were mainly small focal inflammatory areas that could potentially be associated with the site of attachment of the parasite. Similar host responses were also detected from *Merluccius productus* Ayres, 1855 (Meyers et al., 1999). Intestinal cestodes are considered to be non or minimally pathogenic, and in most cases, host tissue damage is elicited by the attachment of the scolex to the host (Eiras et al., 2008). Indeed, despite a single finding of the attachment of the bothria of *C*. *crassiceps* to the intestinal mucosa that caused mild alterations, no other similar changes were detected in any specimen that indicated the previous attachment of this parasite. Granulomas in the stomach were also present in a lower prevalence than in the liver, but were also the result of larval nematode encapsulation, as was described in *Merluccius bilinearis* Mitchill, 1814 (Murchelano et al., 1986). When the parasites (nematodes, cestodes, or digeneans) were found in the mucosa or submucosa of the stomach, no host reaction was observed. On the other hand, rodlet cells have been detected in the gastric mucosa in almost half of the examined specimens. Although their origin and function are still controverted (Manera & Dezfuli, 2004), they have been reported as resident immune cells and considered as biomarkers of exposure to contaminants, including microplastics (Pedà et al., 2016). However, it was already reported in hake that their presence was not associated with higher‐levels of microplastic abundance (Muns‐Pujadas et al., 2023).

The kidney has been highlighted as a target organ of infection for myxosporean parasites (Holzer et al., 2010). Indeed, the presence of coelozoic myxosporean parasites within the renal tubules has been reported in several gadoids, in many other fish species, and in the present study. Sankurathri (1977) described the myxosporean *Conispora renalis* from the renal tubules of *M*. *productus* from the Strait of Georgia, British Columbia, and mixed infections of *Gadimyxa atlantica* and *Zschokkella hildae* were found in cultured *G*. *morhua* (Holzer et al., 2010). In both cases, no major pathological effects were found, as in the current outcomes, despite the high prevalence detected. In fact, it has been reported that many coelozoic myxosporidian parasites coexist with their asymptomatic host without causing severe damage (Colorni & Padrós, 2011).

## 5 CONCLUSIONS

In general, most of the pathologies and associated infections described in the present study were generally focal and produced mild damage. Likewise, parasitic infections typically displayed low intensities, indicating that the host–parasite relationship was in equilibrium. Therefore, no detrimental effects on the organ functionality and health condition of the individuals were expected. Although this study was focused on the juvenile subset of hake, certain pathologies such as *A*. *spinosicanalis* egg infestation in gills should be given special consideration due to their potentially significant impact on the health of individuals and consequently on fisheries. Finally, histopathological studies such as the present one are relevant to gain a more comprehensive understanding of the general health status and pathologies to which wild fish populations are prone, essential for developing effective fishery management strategies.

## AUTHOR CONTRIBUTIONS

L.M.P. drafted the manuscript, performed all analysis and visualizations, and created the figures, with input from all authors. M.C. and F.P. conceived, designed, and supervised the project. S.D. supervised the formal analysis. All authors contributed to the investigation, interpretation of results, and revision of the manuscript.

## FUNDING INFORMATION

This work was supported by the Spanish Ministry of Science and Technology BIOMARE project (CTM2006‐13508‐C02‐01MAR), and by the Spanish Ministry of Science, Innovation and Universities PLASMAR project (RTI2018‐094806‐B‐100). L. M‐P. benefits from an FI‐DGR Ph.D. student grant from the Generalitatde de Catalunya (2022 FI_B 00405).
